# Activity of preclinical and phase I clinical trial of a novel androgen receptor antagonist GT0918 in metastatic breast cancer

**DOI:** 10.1007/s10549-021-06345-x

**Published:** 2021-08-14

**Authors:** Huiping Li, Guohong Song, Qiaoxia Zhou, Ran Ran, Hanfang Jiang, Ruyan Zhang, Yaxin Liu, Jiayang Zhang, Luping Meng, Liandong Ma, Ye Sun, Meiyu Wang, Qingqing Zhou, Honghua Yan, Qianxiang Zhou, Xunwei Dong, Youzhi Tong

**Affiliations:** 1 Key Laboratory of Carcinogenesis and Translational Research (Ministry of Education/Beijing), Department of Breast Oncology, Peking University Cancer Hospital & Institute, 52 Fucheng Rd, Haidian district, Beijing, 100142 People’s Republic of China; 2 Kintor Pharmaceutical Limited, No. 20 Songbei Road, Suzhou Industrial Park, Jiangsu, 215123 People’s Republic of China

**Keywords:** Androgen receptor antagonist, Proxalutamide, GT0918, Metastasis breast cancer, Preclinical data, Phase I clinical trial

## Abstract

**Purpose:**

To evaluate GT0918, a 2nd-generation AR antagonist, for its AR down-regulation activity among breast cancer patients.

**Methods:**

The effect of GT0918 on AR protein expression was evaluated in AR expression breast cancer cells and in breast cancer xenograft model. A 3 + 3 phase I dose-escalation study was launched in Peking University Cancer Hospital. The endpoints included dose finding, safety, pharmacokinetics, and antitumor activity.

**Results:**

GT0918 was demonstrated to effectively suppress the expression of AR protein and the growth of AR-positive breast cancer tumors in mouse xenograft tumor models. All patients treated with GT0918 were at a QD dose-escalation of five dose levels from 100 to 500 mg. The most common treatment-related AEs of any grade were asthenia, anemia, decreased appetite, increased blood cholesterol, increased blood triglycerides, decreased white blood cell count, and increased low-density lipoprotein. Grade 3 AEs were fatigue (2 of 18, 11.1%), aspartate aminotransferase increase (1 of 18, 5.6%), alanine aminotransferase increase (1 of 18, 5.6%), and neutrophil count decrease (1 of 18, 5.6%). Clinical benefit rate (CBR) in 16 weeks was 23.1% (3/13). Among 7 AR-positive patients, 6 can evaluate efficacy, and 2 completed 23.5- and 25-cycle treatment, respectively (as of 2020/1/20). PK parameters showed a fast absorption profile of GT0918 in the single-dose study. GT0918 and its major metabolite reached steady-state serum concentration levels at day 21 after multiple dosing.

**Conclusion:**

GT0918 can effectively inhibit AR-positive breast cancer tumor growth. GT0918 was demonstrated well tolerated with a favorable PK profile. The suitable dose of GT0918 was 500 mg QD and may provide clinical benefits for AR-positive mBC.

## Introduction

The activation of androgen receptor (AR) signaling plays a critical role in driving the initiation and progression of prostate cancer (1). AR-targeted drugs have been approved to treat prostate cancer only. However, accumulated clinical data have supported the potential clinical benefits of AR-targeted therapies for the treatment of patients with breast cancer (2). Recent studies have shown the correlation between AR pathway activation and breast cancer growth, suggesting that AR pathway is a potential therapeutic target for the breast cancer treatment (3–5). Breast cancer can be categorized into different subgroups according to its hormone receptor (HR) and human epidermal growth factor 2 (HER2) status. AR has been identified in 70–90% of ER-positive tumors (6, 7). AR may play different roles in breast cancer progression depending on the HR or HER2 amplification status (8–10). In the ER + AR + breast cancer cells, AR-ligand complex binds to estrogen-related element (ERE) in the nucleus and leads to cell apoptosis (11). On the other hand, in the ER-AR + breast cancer cells, AR complex binds to androgen-related element (ARE) in the nucleus and leads to cell proliferation (12). This hypothesis can explain why patients with ER-positive and AR-positive breast cancer have better prognosis than patients with ER-negative and AR-positive breast cancer (13, 14).

AR has been identified in 10%–35% of triple-negative breast cancer (TNBC) (15–17). The first-generation AR antagonist, bicalutamide, binds to AR-ligand binding domain (AR-LBD) and inhibits its transcriptional activities (18). A phase II clinical trial evaluating bicalutamide in patients with AR-positive, ER/PR-double-negative metastatic breast cancer showed a 6-month clinical benefit rate (CBR) of 19% (15). Enzalutamide, a 2nd-generation AR antagonist, has also been assessed in several studies in TNBC patients and has shown clinical benefits in patients with TNBC treatment, as shown a 16-week CBR of 35% and a 24-week CBR of 29% (16, 19–21). Hence, AR-targeted therapies could be potential treatments for this most aggressive breast cancer subtype. The safety, tolerability, and drug–drug interaction of enzalutamide had been also evaluated, as a mono or in combination with endocrine therapies (ETs), in a recent phase I/Ib clinical trial in patients with advanced breast cancer. Results from this study demonstrated that enzalutamide used as mono or in combination with ETs were well tolerated (22).

Here, we reported the preclinical and clinical data of GT0918 in mBC, including in vitro and in vivo activities of GT0918 in AR-positive metastatic breast cancer models and phase I study results in mBC (safety, efficacy, and pharmacokinetic properties). Our data suggest that GT0918 may have therapeutic benefits in patients with mBC.

## Materials and Methods

### Preclinical studies

#### *Study* cell lines and cell culture

Human Breast cancer lines, MCF-7, BT-474, and MDA-MB-468, were obtained from culture collection of Chinese Academy of Sciences, Shanghai, China. The MCF-7 and MDA-MB-468 cells were cultured in DMEM supplemented with 10% fetal bovine serum and the BT474 was cultured in RPMI1640 supplemented with 10% fetal bovine serum. The cells were cultured in incubator containing 95% air and 5% CO_2_ at 37℃.

### Breast cancer xenograft tumor model

The 6–8 weeks old female BALB/c nude mouse (SPF grade, 18–20 g) was provided by Vital River Laboratory Animal Technology (Beijing, China). Mouse was supplemented with 1.7 mg E2-pellets (90-day release, Innovative Research of America). MCF-7 (1.5 × 10^7^ cells), BT-474 (1.5 × 10^7^ cells), and MDA-MB-468 (1.5 × 10^7^ cells) breast cancer cells were suspended in 200 μL of PBS with 50% Matrigel, and then, the cells were injected orthotopically into the right axial mammary gland to initiate tumor growth. The mouse was then divided (*n* = 8, each group). The mouse was given daily oral dose of GT0918 (5 mg/kg, 10 mg/kg, 20 mg/kg, and 40 mg/kg BID) or MDV3100 (20 mg/kg BID) for 21 days, and 8 mg/kg of cisplatin was injected intraperitoneally once weekly. The body weight and the tumor size were measured twice a week using caliper and tumor volume were calculated according the formula: L × S2 × 0.5, in which L represents the longest diameter and S2 represents the shorter diameter of tumor.

### Clinical study

#### Study design and treatment

This was a phase I, open-label, dose-escalation, and single-center study performed at Peking University Cancer Hospital, China. The study was conducted in accordance with the principles of the Declaration of Helsinki, Good Clinical Practice, applicable laws and requirements. The study protocol was reviewed and approved by the ethics review committee of Peking University Cancer Hospital. All patients were given a written informed consent, and this trial was registered in ClinicalTrials.Gov (NCT04103853).

The primary objective endpoints were the safety, the maximum-tolerated dose (MTD), the recommended dose for expansion (RDE), and dose-limiting toxicities (DLTs) of oral GT0918 in female mBC patients with progression after systemic treatments. The secondary objectives were pharmacokinetics and pharmacodynamics of GT0918 with single and multiple dosages.

The starting dose was 100 mg daily, followed by dose-escalation of 200, 300, 400, and 500 mg. The dose-escalation, in 3 + 3 design, was determined by the safety and tolerability assessments. GT0918 was administered orally once daily, followed by a 7-day off treatment period for single-dose PK analysis of drug elimination. Then, the oral administration of GT0918 was resumed once daily for 28 consecutive days and multiple-dose PK analysis was assessed at the end of first cycle (28 days). The first 28 days on treatment (cycle 1) was defined as DLT period. Patients with an objective response, stable disease, or potential clinical benefit continued the GT0918 treatment until they experienced one of following events of intolerable toxicities, disease progression, or withdrew consent.

#### Study population

Eligible patients were ≥ 18 years old women with an Eastern Cooperative Oncology Group (ECOG) performance status of 0–1 and with histologically confirmed metastatic breast cancer (defined for ER, PR, and human epidermal growth factor receptor by IHC or by in situ hybridization. AR status was suggested to be confirmed). All patients had been progressing after either chemotherapy, hormonal or targeted therapy, or could not tolerate currently standard treatment. Additional eligibility criteria included measurable disease per the Response Evaluation Criteria in Solid Tumors (RECIST 1.1) and adequate hematologic, coagulation, renal, and liver function.

Exclusion criteria included treatments of chemotherapy, radiotherapy, targeted therapy, endocrine therapy, and Chinese traditional medicine therapy in 4 weeks prior the enrollment. Patients were ineligible for enrollment if they had known or suspected central nervous system (CNS) metastases or had a history of seizure, significant cardiovascular disease.

#### Safety assessments

The safety assessments were performed in all patients who received at least one dose of GT0918. Safety was determined by assessment of adverse events (AEs), vital signs, physical examination, 12-lead electrocardiograms, echocardiography, and laboratory tests during the time periods of the first GT0918 dose until 30 days after the last dose. The severity of AEs and abnormal laboratory values were graded using NCI CTCAE version 4.03.

#### PK Assessments

A series of blood samples were collected from each patient for pharmacokinetic analysis at the following timings: PK after single dose: 0 (predose) and at 0.5, 1, 2, 3, 4, 5,6, 8, 12, 24, 48, 72, 96, 120, and 144 h postdose on Day 1, and PK after multiple dosing: 0 (predose) and at 0.5, 1, 2, 3, 4, 5,6, 8, 12, and 24 h postdose on Day 28. In addition, pretreatment samples were collected on days 3, 5, 7, 14, and 21 during cycle 1.

PK analysis for all parameters was performed using Phoenix WinNonlin (Pharsight Corporation, a Certara™ Company, version 8.1) with standard noncompartmental analysis methods. Parameters analyzed included maximum observed plasma concentration (*C*_max_), time of maximum observed plasma concentration (*T*_max_), area under the plasma concentration–time curve (AUC) from time 0 to time of last quantifiable concentration (AUC_0-t_) and AUC from time 0 to 24 h postdose (AUC_0-24_), terminal elimination half-life (T1⁄2), apparent total clearance of the drug (CL/F), and apparent volume of distribution (Vz/F).

### Antitumor activity

Assessment radiographic responses were performed with radiographic scan at baseline, and every 8 weeks or earlier if clinically indicated. Tumor responses were defined by RECIST version 1.1 criteria**.**

### Statistical analysis

All patients receiving at least one dose of GT0918 were included in the analysis. During dose-escalation, the number of patients enrolled in each dose-escalation cohort depended on the observed safety status. MTD was defined as the highest dose tested with at least 6 patients evaluable for toxicity of which fewer than 33% experienced a DLT due to the study drug. For PK analysis, all available PK data from patients receiving GT0918 with adequate concentration distribution were included. Descriptive statistics were used to assess patient characteristics, safety, PK parameters, and anti-tumor activity.

## Results

### The antitumor effect of GT0918 on breast cancer tumors in xenograft models

**Fig. 1:**
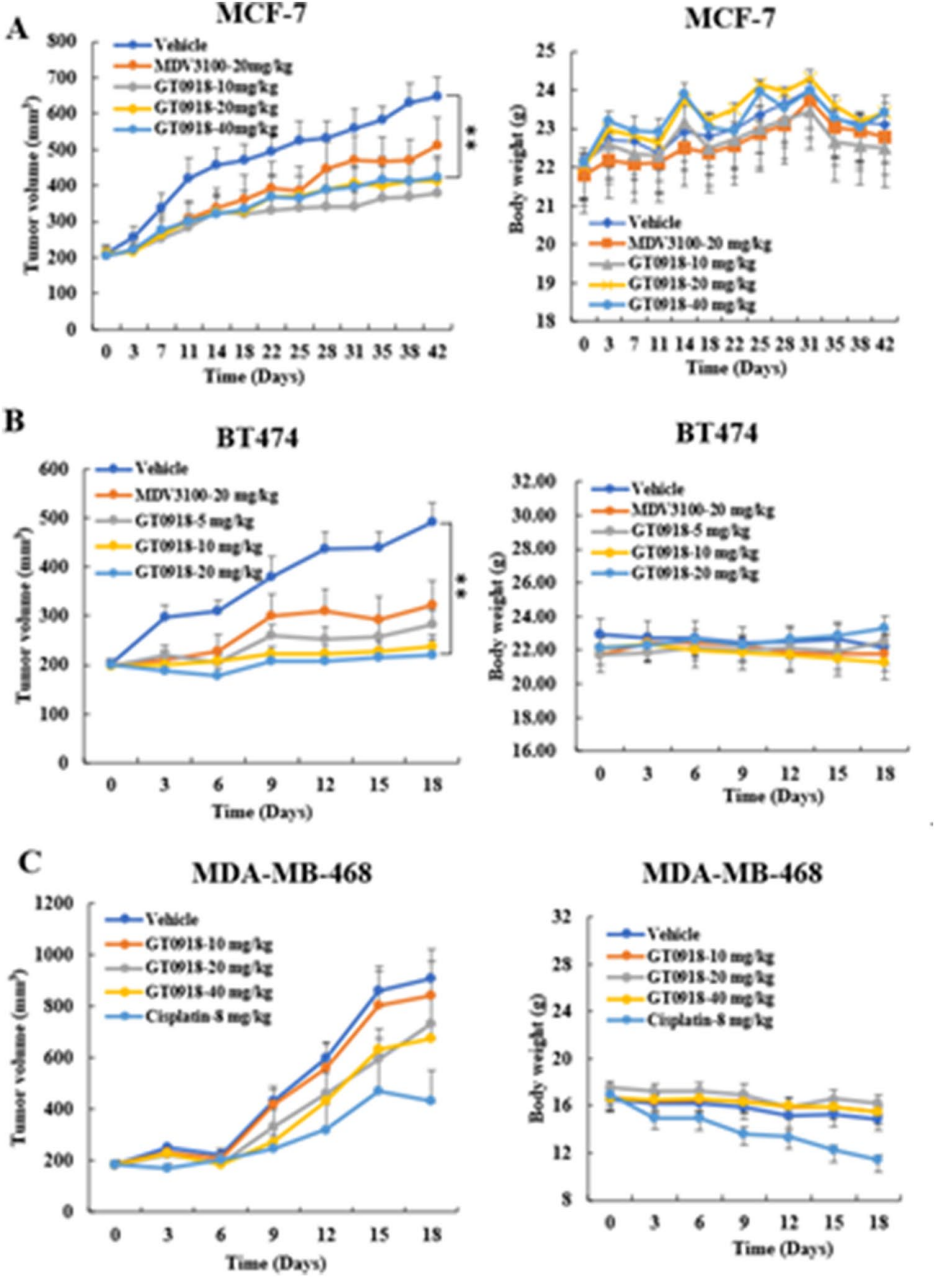
The antitumor effect of GT0918 on breast cancer xenograft model. **A** Effect of GT0918 on MCF-7 xenograft models. **B** Effect of GT0918 on BT474 xenograft models. **C** Effect of GT0918 on MDA-MB-468 xenograft models. The volume of each tumor was measured every 3 days. The average tumor volume in the vehicle, GT0918, MDV-3100, or Cisplatin-treated group was plotted (**p < 0.01, compared to control)

We evaluated the antitumor effect of GT0918 in AR-positive MCF-7 and BT474 breast cancer xenograft tumors. As shown in Fig. 1A, B, GT0918 significantly inhibited the AR-positive MCF-7 and BT474 tumor growth. Compared with MDV3100, GT0918 demonstrated better antitumor activities. In contrast, GT0918 showed no antitumor activity in AR-negative MDA-MB-468 breast cancer xenograft tumors (Fig. 1C). The GT0918-treated groups did not show any body weight change at the tested dosage (Fig. 1A–C), suggesting that GT0918 was not toxic in vivo. These results demonstrate that GT0918 selectively inhibits the growth of estrogen-driven/AR-positive breast cancer tumors with no activity in AR-negative breast cancer tumor model.

### Patient characteristics and disposition

**Fig. 2:**
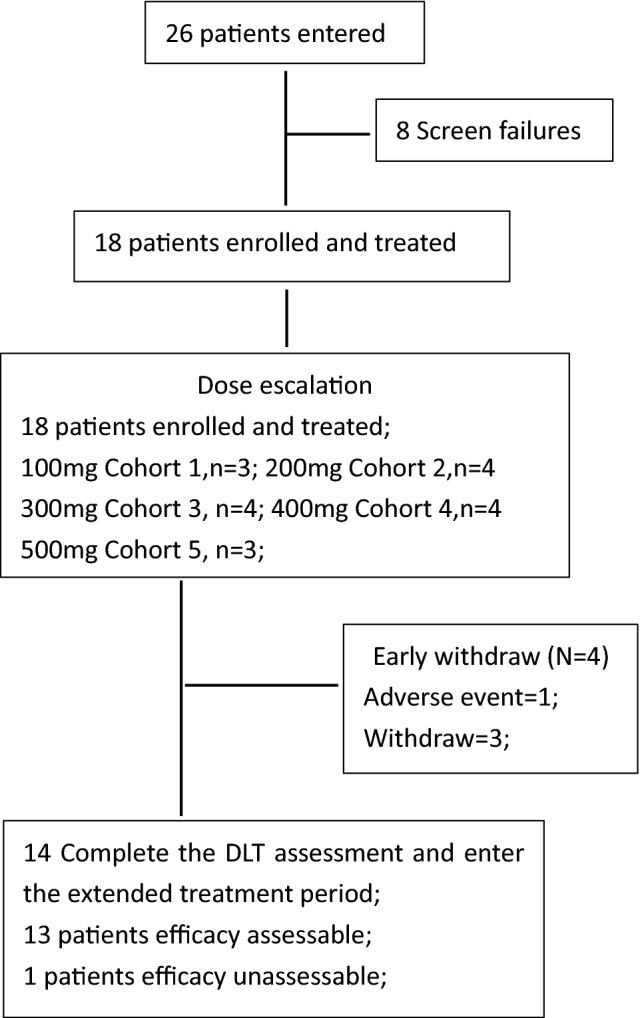
Study diagram

From September 6, 2017 through May 22, 2019, 18 patients were enrolled and treated in the QD dose-escalation. There were 26 patients who entered the study, and 8 failed screening criteria (Fig. 2). Table 1 shows demographic and other baseline disease characteristics of the 18 patients enrolled from the site in China. All the 18 patients had experienced surgery for cancer and systemic therapies previously: chemotherapy (18 patients; 100%), hormonal therapy (12 patients; 66.7%), or targeted therapy (12 patients; 66.7%); the most frequently reported treatments were ≥ 3 regimens (15 patients; 83.3%). A total of ten patients (55.6%) received radiation therapies.

**Table 1:** Patient demographic characteristics

Parameter	Category/Statistic	100 mg (N = 3)	200 mg (N = 4)	300 mg (N = 4)	400 mg (N = 4)	500 mg (N = 3)	Total (N = 18)
Age (year)	N	3	4	4	4	3	18
	Mean	54.3 ( 8.96)	55.8 (11.47)	50.3 ( 5.56)	55.8 (14.41)	49.0 ( 7.55)	53.2 ( 9.48)
	Median	59	60	50	61	50	56
	Min -Max	44–60	39–65	45–56	35–66	41–56	35–66
Fertility, n(%)							
	n	3	4	4	4	3	18
	Yes	1 (33.3%)	1 (25.0%)	4 (100%)	1 (25.0%)	2 (66.7%)	9 (50.0%)
	No	2 (66.7%)	3 (75.0%)	0	3 (75.0%)	1 (33.3%)	9 (50.0%)
	Menarche	0	0	0	0	0	0
	Surgical sterilization	0	0	0	0	0	0
	Menopause	2 (100%)	3 (100%)	0	2 (66.7%)	1 (100%)	8 (88.9%)
	Others	0	0	0	1 (33.3%)	0	1 (11.1%)
Treatment of Primary Tumor							
	Surgery	2 (66.7%)	4 (100%)	3 (75%)	4(100%)	3(100%)	16(88.9%)
	Radiation	3(100%)	3 (75%)	2 (50%)	1 (25%)	1(33.3%)	10(55.6%)
Prior Therapy	Chemotherapy	3 (100%)	4(100%)	4 (100%)	4 (100%)	3 (100%)	18 (100%)
	Hormonal Therapy	3 (100%)	4 (100%)	1 (25%)	2 (50%)	2 (66.7%)	12 (66.7%)
	Targeted Therapy	3 (100%)	0	4 (100%)	3 (75%)	2 (66.7%)	12 (66.7%)
Number of previous regimens for advanced breast cancer	< 3	0	1 (25%)	1 (25%)	1 (25%)	0	3 (16.7%)
	≥ 3	3 (100%)	3 (75%)	3 (75%)	3 (75%)	3 (100%)	15 (83.3%)
AR	N	3	3	3	2	3	14
	AR positive	2 (66.7%)	2 (66.7%)	0	1 (50%)	2 (66.7%)	7 (50%)
HER2 positive		0	0	2 (50%)	0	1 (33.3%)	3 (16.7%)
Triple-negative		0	0	2 (50%)	0	1 (33.3%)	3 (16.7%)
ECOG	0	3 (100%)	4 (100%)	4 (100%)	3 (75%)	3 (100%)	17 (94.4%)
	1	0	0	0	1 (25%)	0	1 (5.6%)

The data cut-off date occurred on January 20, 2020. The median age of patients was 56 (range, 35–66). Most patients (94.4%) had an ECOG PS of 0, and 5.6% had an ECOG PS of 1 at baseline. Overall, 16.7% (3 of 18 patients) had HER2-positive disease, 16.7% (3 of 18 patients) were triple-negative, 77.8% (14 of 18 patients) had HR-positive disease, and 38.9% (7 of 18 patients) had AR-positive disease.

### Dose-escalation

18 patients in total were enrolled in the study: 3 patients in 100 mg, 500 mg group, respectively, and 4 patients in 200 mg, 300 mg, or 400 mg group, respectively. 14 patients were evaluable for DLT (1 experienced progressive disease and 3 withdrew from the study during DLT assessment period). No DLT was observed and MTD has not been reached. Patients complain fatigue and upset but did not reach DLT in 400 mg and 500 mg/day groups. Thus, GT0918 200 mg and 300 mg/day were selected for further testing.

### Safety

All patients were evaluable for safety analysis. Table 2 summarizes all grades of treatment-related AEs occurring in > 10% of patients. The most common non-hematological AEs were asthenia (13 of 18, 72.2%), fatigue (2 of 18,11.1%), anemia (6 of 18, 33.3%), decreased appetite (4 of 18, 22.2%), nausea (3 of 18, 16.7%), constipation (3 of 18, 16.7%), weight loss (3 of 18, 16.7%), increased blood cholesterol (8 of 18, 44.4%), increased blood triglycerides increase (6 of 18, 33.3%), increased aspartate aminotransferase (4 of 18, 22.2%), increased low-density lipoprotein (4 of 18, 22.2%), and increased alanine aminotransferase (3 of 18, 16.7%). The most common hematological AEs were decreased white blood cell count (4 of 18, 22.2%), neutrophil count (2 of 18, 11.1%), and platelet count (2 of 18, 11.1%). These AEs were mainly grade 1–2 and were manageable with supportive care and dose reduction. Grade 3 AEs were fatigue (2 of 18, 11.1%), aspartate aminotransferase increase (1 of 18, 5.6%), alanine aminotransferase increase (1 of 18, 5.6%), and neutrophil count decrease (1 of 18, 5.6%). No treatment-related deaths or DLTs occurred.

**Table 2:** Common AEs (all grades, > 10%; and ≥ grade 3) assessed by investigator as related to treatment by dose

	100 mg (N = 3) n(%)		200 mg (N = 4) n(%)		300 mg (N = 4) n(%)		400 mg (N = 4) n(%)		500 mg (N = 3) n(%)		Total (N = 18) n(%)	
PT name	All	≥ Grade 3	All	≥ Grade 3	All	≥ Grade 3	All	≥ Grade 3	All	≥ Grade 3	All	≥ Grade 3
Asthenia	2 (66.7%)	0	3 (75.0%)	0	2 (50.0%)	0	3 (75.0%)	0	3 (100%)	0	13 (72.2%)	0
Blood cholesterol increased	1 (33.3%)	0	3 (75.0%)	0	2 (50.0%)	0	1 (25.0%)	0	1 (33.3%)	0	8 (44.4%)	0
Blood triglycerides increased	2 (66.7%)	0	3 (75.0%)	0	0	0	1 (25.0%)	0	0	0	6 (33.3%)	0
Anaemia	0	0	1 (25.0%)	0	3 (75.0%)	0	1 (25.0%)	0	1 (33.3%)	0	6 (33.3%)	0
Decreased appetite	0	0	1 (25.0%)	0	1 (25.0%)	0	0	0	2 (66.7%)	0	4 (22.2%)	0
White blood cell count decreased	0	0	0	0	1 (25.0%)	0	0	0	3 (100%)	0	4 (22.2%)	0
Aspartate aminotransferase increased	1 (33.3%)	1 (33.3%)	0	0	0	0	2 (50.0%)	0	1 (33.3%)	0	4 (22.2%)	1 (5.6%)
Low density lipoprotein increased	0	0	2 (50.0%)	0	2 (50.0%)	0	0	0	0	0	4 (22.2%)	0
Alanine aminotransferase increased	1 (33.3%)	1 (33.3%)	0	0	0	0	1 (25.0%)	0	1 (33.3%)	0	3 (16.7%)	1 (5.6%)
Weight decreased	0	0	1 (25.0%)	0	1 (25.0%)	0	0	0	1 (33.3%)	0	3 (16.7%)	0
Nausea	1 (33.3%)	0	0	0	1 (25.0%)	0	1 (25.0%)	0	0	0	3 (16.7%)	0
Constipation	1 (33.3%)	0	1 (25.0%)	0	1 (25.0%)	0	0	0	0	0	3 (16.7%)	0
Neutrophil count decreased	0	0	0	0	1 (25.0%)	1 (25.0%)	0	0	1 (33.3%)	0	2 (11.1%)	1 (5.6%)
Platelet count decreased	0	0	0	0	0	0	2 (50.0%)	0	0	0	2 (11.1%)	0
Fatigue	0	0	0	0	0	0	1 (25.0%)	1 (25.0%)	1 (33.3%)	1 (33.3%)	2 (11.1%)	2 (11.1%)

One patient discontinued the treatment because of AST increase (during the first cycle) which was unlikely treatment-related with GT0918 and probably was related with the disease progression. A total of 5 (27.8%) patients experienced SAEs (considered treatment-related in one patient, including asthenia and decreased appetite)0.1 (5.6%) patient died during this study; the primary cause of death for the patient was disease progression and was considered to be unrelated with the study drug. Patients complaining fatigue were more frequent in the 500 mg group.

### Pharmacokinetics

**Fig. 3:**
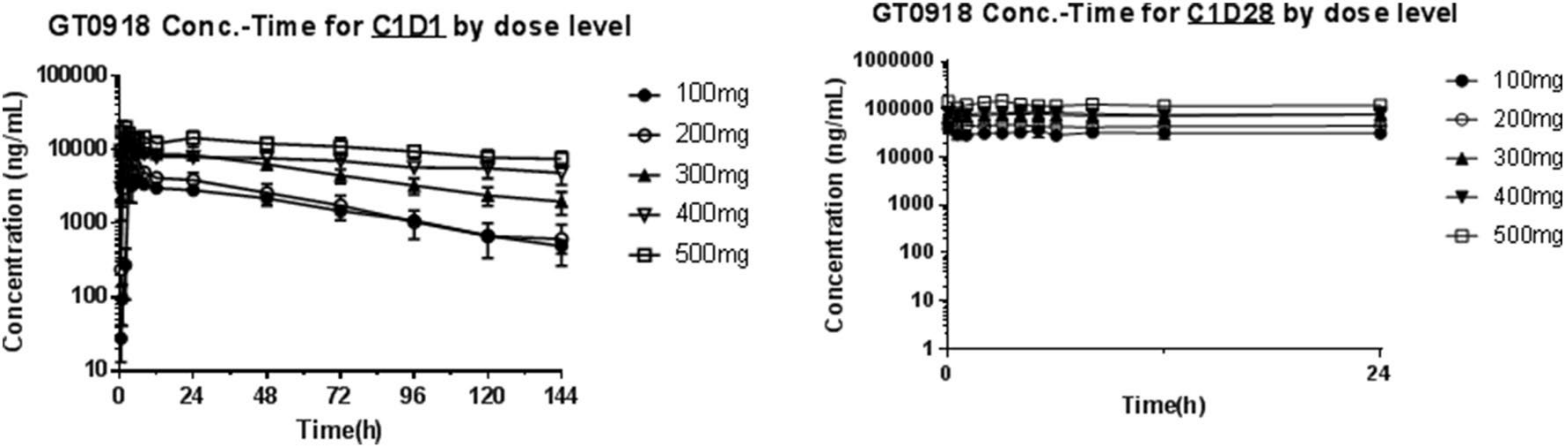
GT0918 Concentration–Time for C1D1/C1D28 by dose level

**Fig. 4:**
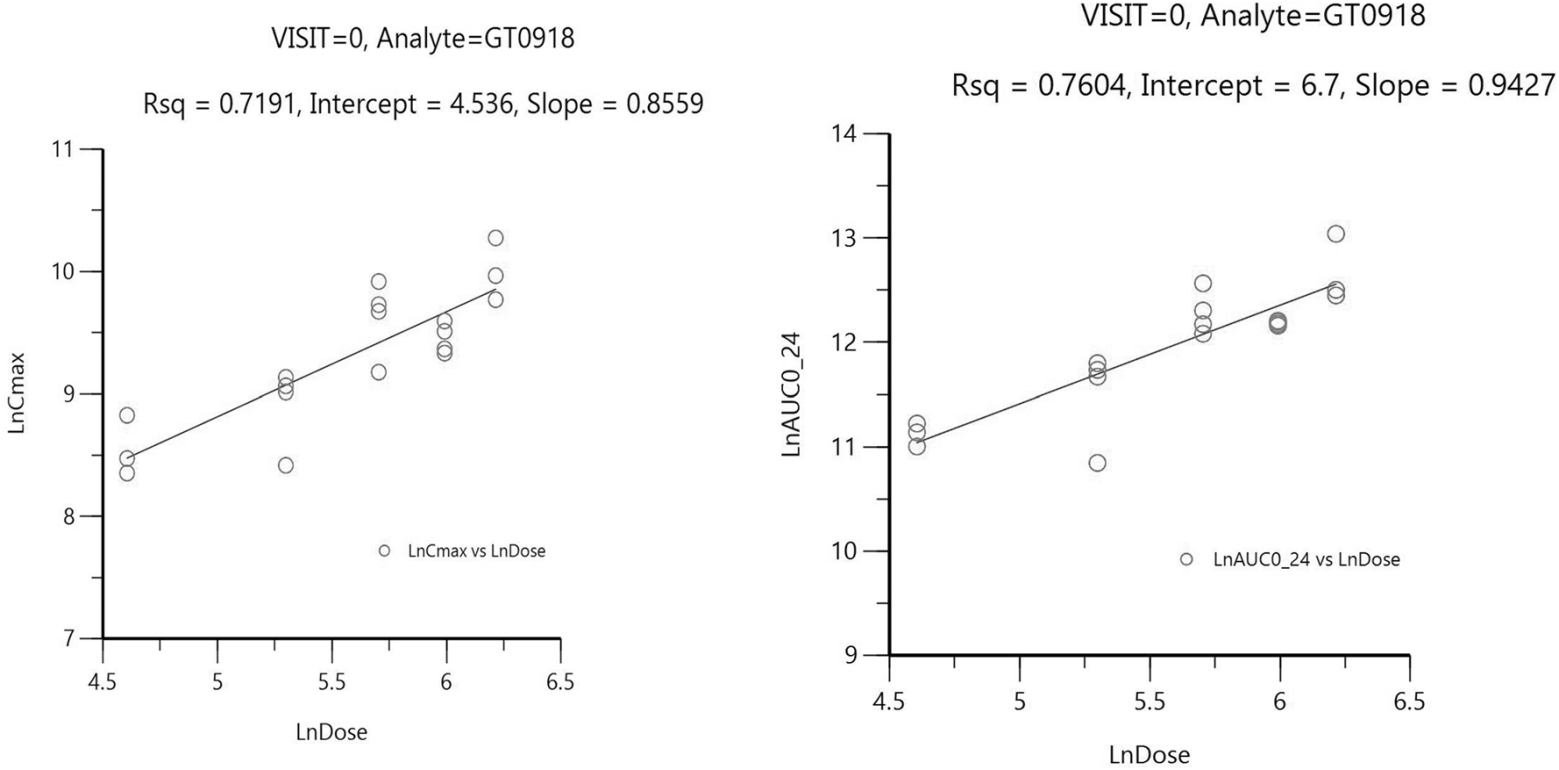
The relationship between exposure and dose proportionally after a single dose

PKs were evaluated in 18 patients, and the PK parameters of GT0918 with single and consecutive administrations are shown in (Tables 3 and 4). The mean plasma concentration vs time profiles following a single dose and multiple doses are shown in Fig. 3. GT0918 was rapidly absorbed after oral administration, as the median time of maximum observed concentration (*T*_max_) was between 1.02 and 3.01 h for single oral dose and between 0.00 and 5.13 h for multiple doses. With a single dosing at 100 mg–500 mg (Fig. 4), *C*_max_ was approximately 5.18, 7.4, 1.51, 1.27, and 2.21 µg/mL, and AUC_0-t_ was 235, 244, 709, 954, and 1470 h·µg/mL, respectively. The steady-state serum concentration level of GT0918 was reached at 21 days in the multiple-dose study. Cmax, ss at Day 28, was 37.3, 50.9, 81.0, 82.7, and 151.0 µg/mL, and AUC_0-t_ was 731, 1040, 1640, 1690, and 2920 h·µg/mL, respectively. Drug exposure parameters including the area under the concentration–time curve (AUC) and maximum concentration (*C*_max_) were increased with dose proportionally after a single dose and multiple doses ranging from 100 to 500 mg. The ratio of AUC from 0 to 24 h postdose (AUC_0-24_) between day 28 after daily dosing and day 1 after single dosing was from 8.35 to 11.0, suggesting a drug accumulation after multiple dosing. Terminal half-life (T_1/2_) ranged from 25.1 to 97.4 h across the dose levels for single administration. Mean apparent clearance values (CL/F) ranged from 0.217 to 1.1 L/h for single administration and from 0.10 to 0.2 L/h for consecutive administration, suggesting that the elimination of GT0918 was slow.

**Table 3:** GT0918 steady-state pharmacokinetics following 28 days of once-daily administration (geometric Mean ± CV%) *Tmax: presented as Median (Min, Max)

PK parameter	Cohort 1 100 mg	Cohort 2 200 mg	Cohort 3 300 mg	Cohort 4 400 mg	Cohort 5 500 mg
*C*_max_ (μg/ml)	37.3 40	50.9 17.0	81 57.3	82.7 64.1	151 13.1
*T_max_ (h)	3.05 (0.00,4.97)	0.00 (0.00, 3.98)	3.03 (0.00, 5.03)	5.13 (1.88, 8.12)	1.50 (0.00, 3.00)
Cavg (μg/ml)	30.3 37.6	43.4 25.3	68.3 59.2	70.9 64.0	121 16.5
AUC_tau_ (h*ug/ml)	728 37.6	1040 25.3	1640 59.2	1700 64.0	2910 16.5

**Table 4:** GT0918 pharmacokinetics parameters following a single dose administration (geometric mean (CV%))

	*t*_1/2_ (h)	*T*_max_ (h)	*C*_max_ (µg/mL)	AUC_0-t_ (h*µg/mL)	*V*_z_/F (L)	CL/F (L/h)
100 mg (*n* = 3)	46.5 (15.1)	3.00 (2.97, 6.00)	5.18 (25.0)	235.0 (14.0)	25.1 (4.1)	0.374 (18.5)
200 mg (*n* = 4)	25.1 (78.4)	3.01 (2.02, 3.03)	7.40 (34.0)	244.0 (103.5)	27.8 (50.5)	0.766 (114.4)
300 mg (*n* = 4)	57.6 (24.6)	2.99 (1.00, 3.05)	15.10 (32.4)	709.0 (29.0)	28.8 (17.2)	0.346 (40.6)
400 mg (*n* = 4)	77.9 (57.2)	1.98 (1.97, 4.03)	12.70 (12.4)	954.0 (18.5)	34.2 (13.9)	0.305 (40.9)
500 mg (*n* = 3)	97.4 (20.3)	1.02 (1.00, 2.00)	22.10 (25.9)	1470.0 (39.5)	28.5 (31.6)	0.203 (50.3)

*T_max_ presented as Median (Min, Max) *T*_max_ time to peak concentration, *C*_max_ peak concentration, *AUC*_*0-*t_ area under the curve from time 0 to last quantifiable concentration, *V*_*z*_*/F* apparent volume of distribution, *CL/F* apparent total clearance, *CV* coefficient of variation

### Antitumor activity

**Fig. 5:**
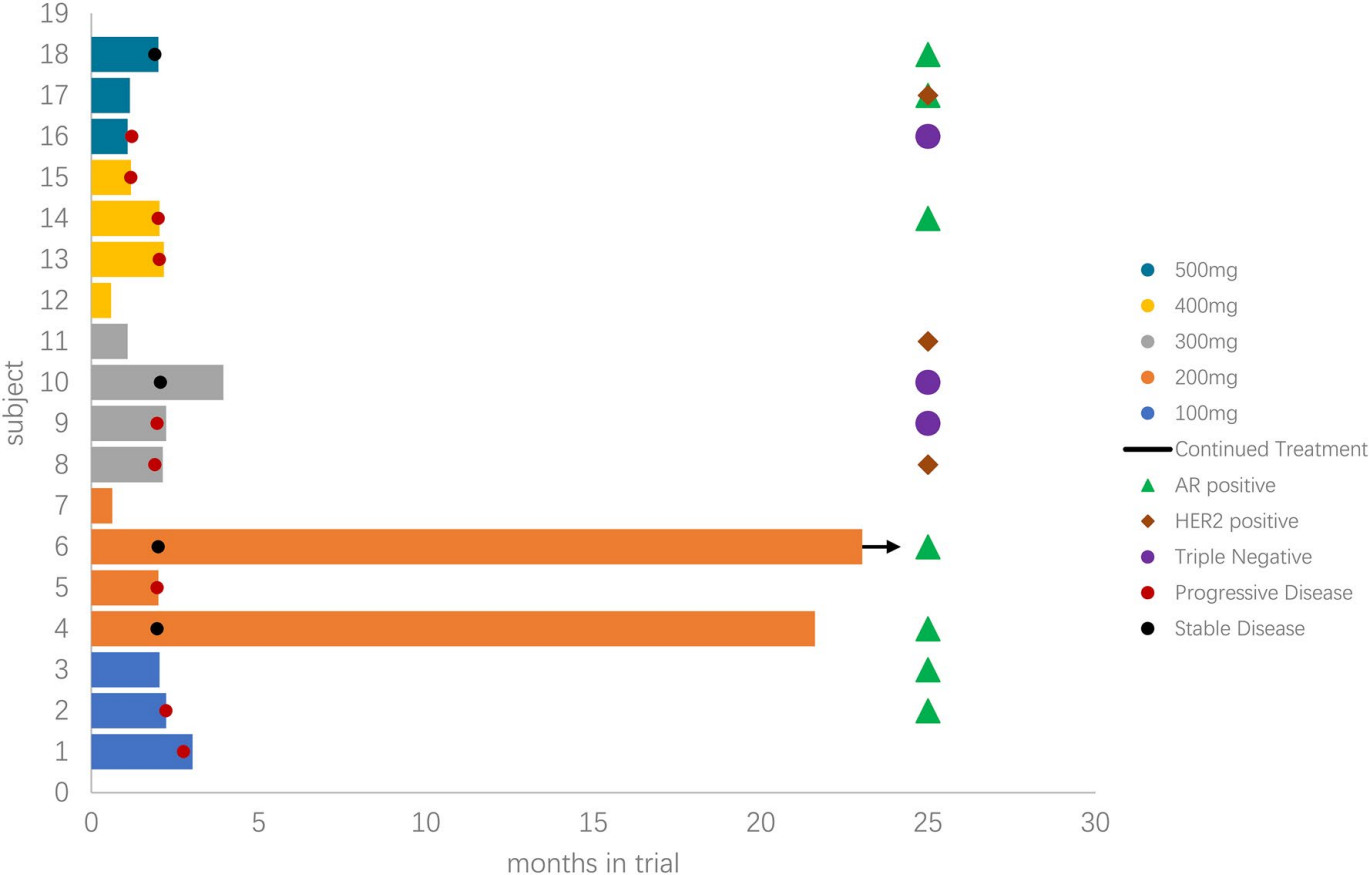
Swim plot

In the dose-escalation phase, 18 patients were exposed to study drug with doses ranging from 100 to 500 mg QD on a 28-day cycle. All patients progressed from more than two lines of therapies, and 83.3% (15/18) patients progressed three lines or more. There were 13 patients can evaluate efficacy, four patients obtained SD (Fig. 5), 3 of them last more than 16 weeks; clinical benefit rate (CBR) in 16 weeks was 23.1%. Out of the seven confirmed AR + patients, three got response: 1 was in 500 mg, 8 weeks treatment, and withdrew study because of fatigue; 2 in 200 mg dose group; those two patients’ lesions were obtained reduced SD, one patient was lung, pleural fluid, and bone metastasis, had 1 line chemotherapy (Docetaxel), 3 line endocrine therapy (Toremifene, Exemestane, Fulvestrant), she obtained 23.5 cycles; another one was liver and lung metastasis, experienced 1 line chemotherapy (docetaxel), 2 line endocrine therapy (Exemestane, Fulvestrant), she obtained 25 cycles treatment, and continue treat as of January 20, 2020. The following charts (Fig. 5) illustrated the treatment cycles of patients.

## Discussion

AR has been identified in 60–80% breast cancer patients (10). Accumulated data have demonstrated the important role of AR-signaling in breast cancer tumorigenesis and disease progression (8, 23). Therefore, it is an unmet medical need for clinical development of AR therapies for patients with AR + breast cancers. However, it has not been successful in the clinical studies of AR pathway inhibitors in clinical up to date. The role of AR-signaling may be different among subtypes of breast cancer. Therefore, it is very critical to develop new strategies for clinical studies of AR-targeted therapies for breast cancer and to identify the subgroup that is most likely to benefit from AR-targeting therapies. Here, we disclosed GT0918, a 2nd-generation AR antagonist, for breast cancer treatment.

Our preclinical data demonstrated that GT0918 inhibited the growth of AR-positive breast cancer xenograft tumors. These data support a clinical testing of GT0918 in AR-positive breast cancer tumors.

This phase I study was the first investigation of GT0918 in women with breast cancer. A total of 5 dose levels were tested (100 mg, 200 mg, 300 mg, 400 mg, 500 mg) in a total of 18 patients. The primary objectives were to characterize the PK properties of GT0918 and to assess the safety and tolerability of GT0918 in order to support further clinical studies in patients with breast cancer.

Results from this clinical study in breast cancer patients proved GT0918 to be well tolerated at a maximum dose of 500 mg/day. And the pharmacokinetics of single- and multiple-dose GT0918 in women with breast cancer was found to be similar to the pharmacokinetics of GT0918 in men with prostate cancer (24). Oral absorption of GT0918, single or multiple doses, were rapid and dose-independent peak concentrations of GT0918 were achieved from 1 to 3 h postdose. The mean apparent volume of distribution (V/F) of GT0918 in patients ranged from 25.1 to 34.2 L after a single dose indicating limited extravascular distribution. After a single- or multiple-dose, pharmacokinetics of GT0918 was proportionally correlated with the doses ranging from 100 to 500 mg. The mean apparent clearance of GT0918 ranged from 0.203 to 0.766 L/h, which was approximately 1.5% of the liver plasma flow rate (48.7 L/h), and this suggested that GT0918 is a low extraction ratio drug.

This study demonstrated that GT0918 had an acceptable safety profile in patients with advanced breast cancer, with mild and moderate level of AEs. The most common drug-related adverse events (> 15%) were asthenia (72.2%), increased blood cholesterol (44.4%), increased blood triglycerides (33.3%), anemia (33.3%), decreased appetite (22.2%), decreased white blood cell count (22.2%), increased aspartate aminotransferase (22.2%), increased low-density lipoprotein (22.2%), increased alanine aminotransferase (16.7%), weight lost (16.7%), nausea (16.7%), and constipation (16.7%). The adverse events (≥ 3 Grade) were fatigue (11.1%), increased aspartate aminotransferase (5.6%), increased alanine aminotransferase (5.6%), and decreased neutrophil count (5.6%).

This incidence of fatigue is similar to the reported fatigue AE in other studies of AR inhibitors. Importantly, no seizures were observed yet at any dose level.

Preliminary signs of clinical activity of GT0918 in patients with AR + mBC were observed. CBR in 16 weeks was 23.1% (3/13); among 7 AR + patients, 6 can evaluate efficacy; among AR + patients, CBR in 16 weeks was 33.3% (2/6) (Fig. 5). Out of the seven confirmed AR + patients, one patient had completed treatment of 23.5 cycles and another patient was still under treatment after completed cycle 25 as of January 20, 2020. Because of the limited sample size of this clinical trial and unrestricted androgen receptor status, one cannot conclude that the patients with high AR expression would have an improved clinical outcome with AR inhibitor treatment. However, it would be interesting to evaluate the efficacy of GT0918 in the expanding clinical studies with AR + patients.

According to the safety, tolerability, and pharmacodynamics analysis, GT0918 shows preliminary signs of clinical activity in AR + breast cancer, thereby warranting further evaluation. Because we did not see 16 weeks response in the 400 mg and 500 mg cohort, two longer duration treatment patients both in 200 mg group obtained 23.5 and 25 cycles and one of the patient had 4.5 cycles in 300 mg group (Fig. 5), and so we consider that lower dose can reach the effective level of treatment. The same phenomenon of GT0918 was also found in prostate cancer (24). As the PK results showed that the drug exposure was increased with dose after a single dose and multiple doses ranging from 100 to 500 mg, especially the drug showed linear pharmacokinetics in the dose range of 100–300 mg (Table 3, Fig. 3). Following a single dose, the values of *C*_max_ at 200 mg, 300 mg, and 400 mg dosage were 7.4 μg/mL, 15.1 μg/mL, and 12.7 μg/mL, respectively (Table 4, Fig. 3), and after consecutive 28 days administration, the Cmax were 50.9 μg/mL, 81.0 μg/mL, and 82.7 μg/mL (Table 3, Fig. 3). The results indicating that when the dosage was increased from 200 mg, 300 mg to 400 mg, there was no significant increase in drug exposure. At higher dosage, although the exposure increased, it was less than the proportion, indicating that the exposure seems to saturate. The recommended doses for expansion are 200 mg and 300 mg daily, which are similar to GT0918 in prostate cancer.

Based on these results, an expanded/phase Ib and Ic trials have been initiated in patients with AR-positive mBC, being progressed from multiple treatment lines.

## Conclusion

GT0918, the 2nd-generation AR antagonist, has potential antitumor effect in AR-positive breast cancer. GT0918 is well tolerated with a favorite PK profile and reveals a prospective antitumor activity in AR-positive mBC; the suitable dose of GT0918 was 500 mg QD; the presented results indicate a promising alternative treatment for AR-positive mBC patients in the future.
